# Analysis of Stress-Strain State for a Cylindrical Tank Wall Defected Zone

**DOI:** 10.3390/ma15165732

**Published:** 2022-08-19

**Authors:** Nurlan Zhangabay, Bayan Sapargaliyeva, Ulanbator Suleimenov, Khassen Abshenov, Akmaral Utelbayeva, Alexandr Kolesnikov, Kanat Baibolov, Roman Fediuk, Dinara Arinova, Bolat Duissenbekov, Azamat Seitkhanov, Mugahed Amran

**Affiliations:** 1 Department of “Construction and Construction Materials”, M. Auezov South Kazakhstan University, Av. Tauke Khan, 5, Shymkent 160012, Kazakhstan; 2 Abai Kazakh National Pedagogical University, Almaty 050010, Kazakhstan; 3 Department of “Architecture”, M. Auezov South Kazakhstan University, Av. Tauke Khan, 5, Shymkent 160012, Kazakhstan; 4 Department of “Mechanics and Mechanical Engineering”, M. Auezov South Kazakhstan University, Av. Tauke Khan, 5, Shymkent 160012, Kazakhstan; 5 Department of “Chemistry”, M. Auezov South Kazakhstan University, Av. Tauke Khan, 5, Shymkent 160012, Kazakhstan; 6 Department of “Life Safety and Environmental Protection”, M. Auezov South Kazakhstan University, Av. Tauke Khan, 5, Shymkent 160012, Kazakhstan; 7 Peoples Friendship University Named after Academician A. Kuatbekov, Str., Tolebi n/n, Shymkent 160012, Kazakhstan; 8 Polytechnic Institute, Far Eastern Federal University, 690922 Vladivostok, Russia; 9 Peter the Great St. Petersburg Polytechnic University, 195251 Saint Petersburg, Russia; 10 Department of Civil Engineering, College of Engineering, Prince Sattam Bin Abdulaziz University, Alkharj 16273, Saudi Arabia; 11 Department of Civil Engineering, Faculty of Engineering and IT, Amran University, Amran 9677, Yemen

**Keywords:** tank, stress concentration, defects, dent, dimensionless parameters, experimental study, numerical method, modeling

## Abstract

In the study, experimental and theoretical studies were carried out to assess the influence of the shapes of dents in the tank wall on the stress-strain state of the defect zone. By testing fragments of a cylindrical tank, it was found that the most appropriate expression is (5), which could take into account the leaching of the tank wall, resulting in a decrease in the stress concentration index. At the same time, during theoretical studies in this paper, it was found that polynomials determined the stress concentration coefficient, where the obtained analytical expression data were compared with the data determined numerically in the ANSYS program, and it was found that the spread was from 2% to 10%. According to the results of a numerical study of the stress-strain state of the dent zone in the tank wall, graphical dependences of the stress concentration coefficient on the dimensionless depth of the dent for various values of the dimensionless radius of the dents and do not exceed 2% of the indicators that are obtained. At the conclusion of the experimental and numerical studies, a conclusion was made about the degree of influence of the geometric dimensions of the dents on the stress concentration index.

## 1. Introduction

The construction and maintenance to ensure the operational condition of existing energy facilities in the form of tanks for storing oil and oil products [1,2,3,4,5], as well as the assessment of the strength, durability and stability of steel vertical cylindrical tanks leads to the constant improvement of existing methods, materials and structures [6,7,8,9]. The practice of using vertical cylindrical tanks shows that the most likely causes for the initiation and development of their destruction are dents located in the tank wall, which are unpredictable and understudied from the point of view of the stress-strain state [10,11,12,13,14,15,16].

A review of the literature data showed that in [17] only the issue of repairing dents with carbon fiber reinforcement was raised in order to restore the equipment to its initial bearing capacity. The issue of corrosion, taking into account the defect, was studied only on linear structures [18]. The work described in [19] provides a method for determining elastic deformations on a section of the wall of a steel vertical tank which take the form of dents, using minimal information about their parameters, where dents of the correct shape, i.e., those which form of an ellipse, were considered. The restrictions that were introduced on the defective character of the “Dent” type in steel tanks, according to the regulatory documents for the design and operation of steel tanks in different countries, are given in [20]. However, that which is not shown in the literature is the issue of durability and the absence of the stress concentration coefficient’s mathematical dependence on the geometric dimensions of complex dents in the dent zone, which take into account the steel vertical tank’s continuous and variable wall thickness. Works by the authors’ of [21,22] are mainly devoted to the problem of the vertical steel tank shell’s durability during the corrosion process, which is caused by the thinning of the walls. The research methodology, taking into account the geometric dimensions and defect parameters, can positively complement the study described in [23] which concerns the seismic resistance of linear structures in the form of a large diameter pipeline. So, according to the authors of [24], corrosion tends to worsen in the defect places, therefore, research in the field of predicting the durability of the shell structure that is under consideration, when taking into account complex local defects, can positively supplement the work that was considered earlier. In particular, we are talking about works on the study of the structure’s durability, taking into account the corrosion process. This will also help to solve a number of problems in the field of corrosion, with consideration for the difference in the shell wall thickness and the corrosion effect in this aspect. The paper, [25], presents the results of studies from the ANSYS program which describe the stress-strain state of a vertical steel tank, where reinforcement of the structure was proposed as a solution to avoid stress concentration, and the forms of the dents and the causes of their formation are not investigated. However, there are no experimental studies on models or full-scale objects. In the work described in [26], the issue of changing the stress-strain state of a steel vertical tank, with a volume of V = 10,000 m^3^, for storing commercial and substandard oil was considered, taking into account only the displacements and deformations that formed as a result of displacement. However, the question of the nature and shape of these deformations was not considered, which is also relevant. In the work described in [27], the issue of the stress-strain state of steel structures during the propagation of deformations in the form of cracks was considered, where only the issue of the length of the defect was analyzed, and no other geometric characteristics were considered. In paper [28], the oscillatory, sliding contact between a rigid, rough surface and an elastic-plastic half-space was considered in the context of numerical modeling. The paper, [29], presents a method for calculating the J-integral and T*integral in the framework of the EFG method. The proposed method is applied to both a stationary crack problem and a stable crack growth problem. In the study described in [30], through static analysis, the resultant force of particles in rock fissures is extruded by the rock on both sides. The study described in [31] attempted to simulate a girth welded pipeline with various depths and lengths of corrosion in order to compare it with offshore pipeline design manuals. Based on the numerical results, the influence of corrosion defect parameters on the strength of girth welded pipelines were investigated. The paper, [32], investigates a method for assessing the ability to destroy a real pipeline subject with large plastic deformation.

The considered works’ results show that there are general issues of predicting the durability of steel shell structures, which significantly affect further operations. However, this does not make it possible to accurately assess the effect of various types of defects that take the form of complex dents, or more accurately determine the service life of steel shell structures in terms of the stress concentration in the dent zones. 

In this regard, the aim of the research is an experimental and theoretical assessment of the stress-strain state of the cylindrical tank wall’s dent zone and the identification of the stress concentration’s dependence on the geometric dent parameters and the tank dimensions, which will subsequently make it possible to predict the service life of vertical steel tanks when taking into account defects in the form of dents. The research results can be used in the design and construction of steel vertical cylindrical tanks and when predicting the durability of these structures.

## 2. Materials and Methods

One of the main tasks of modeling thin-walled cylindrical structures is to achieve maximum correspondence between the laws of mechanical processes occurring in the model and the full-scale structures. When modeling thin-walled structures, the similarity between the structure and the model may be violated due to the different overall dimensions of the shell body and wall thickness. This phenomenon is associated with the violation of mechanical similarity and is called the scale effect, which is especially evident when modeling large-sized reservoirs. 

The most important prerequisites for reducing the influence of the scale effect are to ensure high quality of work in the manufacture of models, and the establishment of similarity in the stochastic formulation of the question. Since the theoretical solution for determining the similarity criteria is made in this formulation, the manifestation of the scale effect will not impose fundamental restrictions on the study of the tank structure’s stress-strain state when employing modeling. 

**Figure 1:**
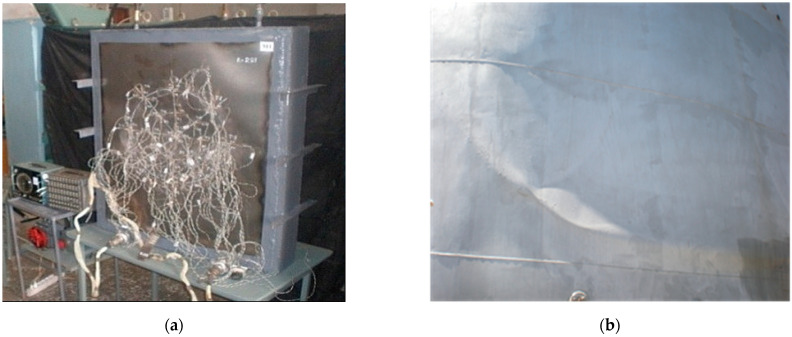
View of the dent on the model (**a**) and full-scale object (**b**) [33,34].

According to the purpose of experimental studies, to study the features of the tank body’s dent zone stress-strain state, models of the tank body fragment with various dent shapes and geometric dimensions were made. A general view of the models that were made for testing and their main components are presented in Figure 1. 

The model was manufactured, and it represents a fragment of the body of a cylindrical shell with a radius of 2280 mm. Its overall dimensions were 1200 × 1000 × 162 mm. Its frame was formed from channel No. 16. Its body was made from sheet steel with a grade VSt3sp and was 1.0 mm thick, while the model body material’s chemical composition was determined using the gas-volumetric method [35], and its mechanical characteristics were determined by employing the standard testing of samples according to the study described in [36].

**Figure 2:**
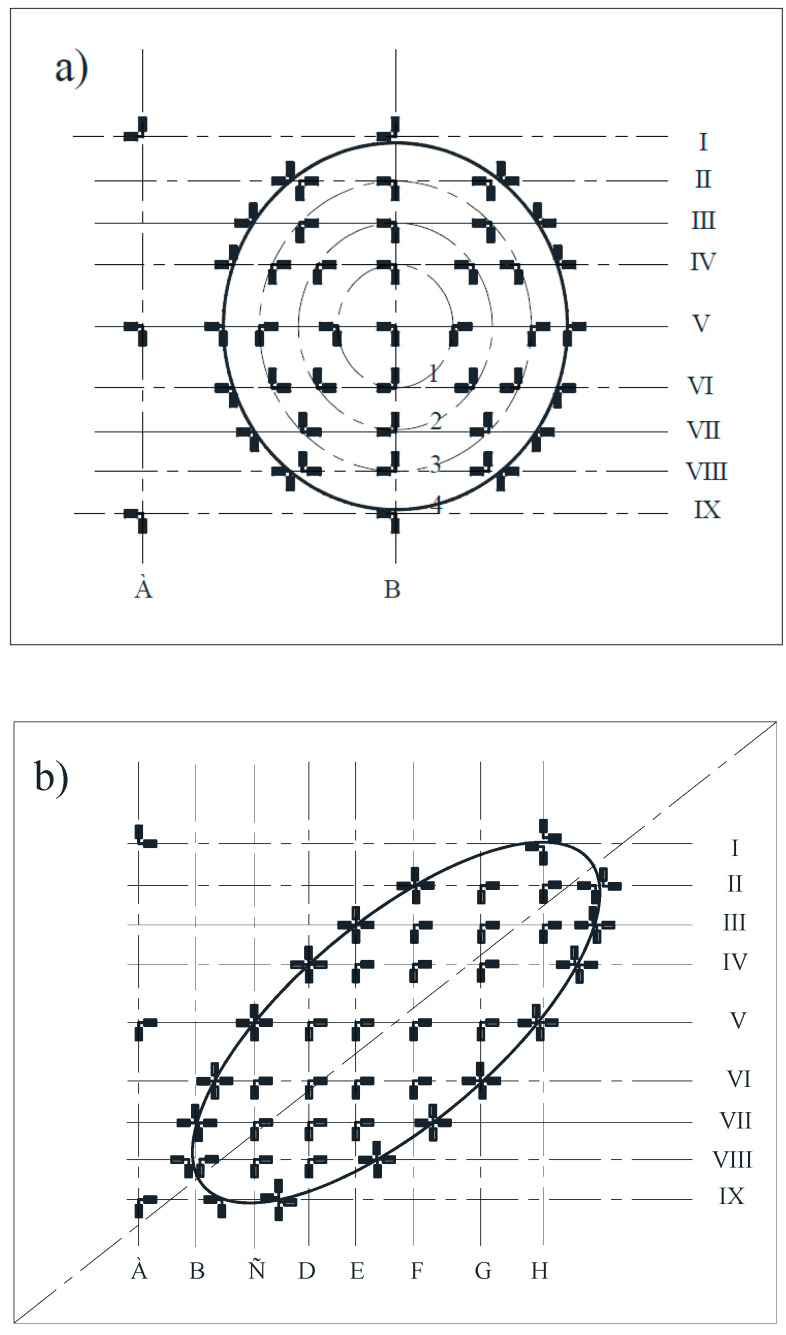
Layout of load cells in the zone of spherical (**a**) and ellipsoidal dents (**b**).

There were two fragments made of the shell body with two modeled dents of a spherical and elliptical shape on one of these models, as shown in Figure 2. 

The main geometric dimensions of the dents on the model wall are presented in Table 1.

On model M1, dents were modeled by crushing the model wall’s surface with special stamps. On the surface of the wall of model M2, these were devised by selecting a method and scheme of welding the end parts of the wall sheet and were produced by forming welding deformations. 

The models were placed on a special frame, the racks of which were embedded in the concrete floor. 

To simulate overpressure, a fitting with a diameter of 15 mm was placed on the model’s roof to ensure there was an air supply for a compressor and a branch pipe for the installation of a spring pressure gauge. The model’s tightness was tested with compressed air at a pressure of 0.02 kg/cm^2^. 

In order to place dial indicators on the model, rigid strips of metal were welded onto the end part, where rods were installed with special clamps to fix the indicators. 

The choice of methodology for conducting experiments on models of a vertical cylindrical tanks was determined by a set of rules and the application of certain principles for testing. These principles consist of: the reproduction of operational impacts and the choice of design parameters that were as close as possible to the real conditions, the use of equipment, instruments and apparatus that correspond with the goals and tasks set for experiments, and the use of appropriate methods for recording and processing experimental data and the form of their presentation. 

The choice of measuring instruments, equipment and primary converters was made by taking into account the expected values of the experimental data, the conditions for the experiment and were based on the recommendations of other works [36,37]. 

The load generated from the internal pressure during the tests was simulated by injecting air into the shell using a compressor, which allowed for the maintenance of a pressure of 8 MPa. The pressure inside the test model was controlled using a spring pressure gauge with a division value of 0.02 MPa. 

Since the main goal of the experimental studies was to study the nature of the stress-strain state in the dent zone and to establish the stress concentration coefficients, it became necessary to determine the stresses at the characteristic points of the dent zone and measure the deformation during the process of loading the model with internal pressure. 

To register relative strains at characteristic points of the dent zone and to study the stress state of the defect zone, a strain-measuring method was chosen [38]. 

**Figure 3:**
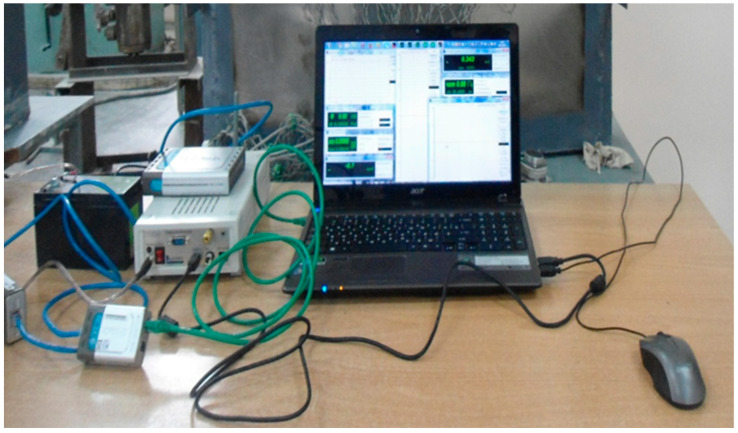
Strain gauge station, ZET 017–Tb.

Single-element, loop paper-based strain gauges with a base of 10 and 20 mm were used as primary converters for measuring relative strains (Figure 2). The strain gauge station, ZET 017–Tb (Spectrum Analyzers, Moscow, Russia) [39], was adopted as the secondary recording equipment (Figure 3).

The strain gauges were placed in such a way that it was possible to measure the deformation in two orthogonal planes: hoop and longitudinal to the axis of the shell in places of characteristic bending depths and changes to the wall shape (Figure 2). 

The strain measurements were carried out by placing a rosette of strain gauges on the outside, along the symmetrical axes of the dents to determine the hoop and longitudinal surface deformations, which was in accordance with the recommendations [40]. The hoop and longitudinal stresses were calculated using the following formulae

(1) $$\sigma_{1}=\frac{E}{1-\mu^{2}}\left(\varepsilon_{1}+\mu \varepsilon_{2}\right)$$

(2) $$\sigma_{2}=\frac{E}{1-\mu^{2}}\left(\varepsilon_{2}+\mu \varepsilon_{1}\right)$$

where *E* = 2·10^5^ Mpa, elasticity modulus; and *μ* = 0.3—Poisson’s ratio. 

**Figure 4:**
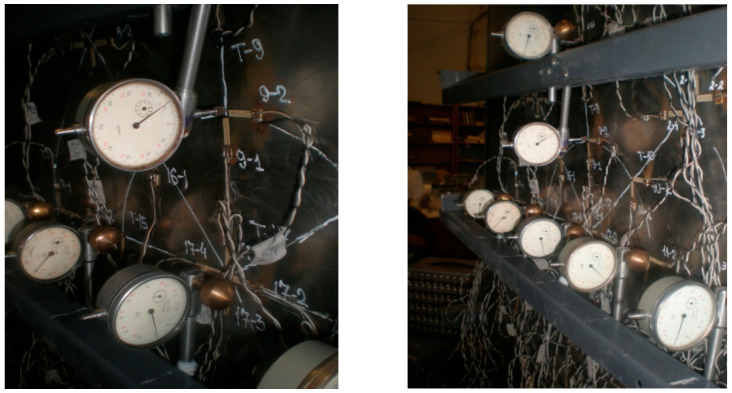
Ich-04 displacement indicators, fixed on special brackets.

ICh-04 indicators were fixed on special brackets, as shown in Figure 4.

In the cylindrical shells with dents in the body, the phenomenon of the walls snapping out in the dent zone was observed, which was accompanied by a decrease in the dent depth and its straightening. It is not possible to establish using calculation the conditions under which the wall snapped out. 

To study this phenomenon and take into account the irreversible decrease in the dent depth in the process of loading the model, the stepwise loading of the models with a pressure of 2 kPa was adopted, and then stepwise unloading was performed with exposure at each step for 2–3 min and steady-state displacements were measured. When the phenomenon of the model wall snapping out during the air injection by the compressor was detected, the compressor was turned off, the valve was closed, and reports were taken from the strain gauges and displacement indicators as well as readings from the pressure gauges. 

Taking into account the maximum possible loads that could be applied to the tank design model, the maximum pressure in the model was limited to 8 kPa. 

In the process of theoretical and experimental study of the stress-strain state of the wall in the stress concentration zone, the initial dent depths were taken as their steady-state values, as shown in Table 2. 

After reaching the maximum internal pressure in the model, stepwise unloading was carried out with the registration of the readings of the pressure gauge and displacement indicators and the removal of reports on strain gauges. 

The obtained test results were processed in compliance with the procedures and methods for solving practical problems of statistics when the measured values are random and distributed according to the normal law, in accordance with the study in [41].

The stress concentration coefficients at the characteristic dent zone points were determined by the ratio of the maximum stresses in the dent zone in relation to the nominal stress at the characteristic point of the model wall’s momentless zone according to the formula:

(3) $$k=\frac{\sigma_{\max}}{\sigma_{n}},$$

where *σ*_max_ —the maximum stresses at the characteristic dent zone point; and *σ_n_* —the nominal stresses in the wall.

To solve the problem of theoretical research, it is most effective to use the finite element method, which is implemented using the ANSYS software package. 

Numerical studies of the stress-strain state of tank structures, like any other structures, require the replacement of real structures with a mathematical model. The selected mathematical model should have the basic properties of the calculated full-scale structure and at the same time be quite simple and suitable for engineering calculations. 

The choice of a calculation scheme for the numerical simulation of the stress-strain state of a vertical cylindrical tank wall that has a dent with a known defect topography is not a difficult task when using the ANSYS finite element packages. The software package allows the calculator to create and calculate models of any complexity and create an optimal design scheme by switching from a more complex and realistic structure to a computationally simpler scheme. The methodology of this study is described in detail in [42]. 

It can be concluded that in the case of the dent geometry, nothing is known except their basic dimensions, and if the dent shape is complex, it is advisable to use a spherical scheme for idealizing the dent shape within the calculations. 

The results of these experimental and theoretical studies will serve as the basis for determining the mathematical dependence of the stress concentration coefficient on the geometric dimensions of the dent and substantiate the methodology that has been developed on its basis for assessing the strength and residual life of tanks with defects that take the form of dents in the wall. 

## 3. Theory and Calculation of Research

### 3.1. Experimental Studies of the Stress-Strain State of the Dent Zone in the Tank Wall

Analytical methods for assessing the stress-strain state in the zones of the defects and imperfections that cause high stress concentrations contain significant assumptions due to the conventionality of the accepted design schemes [43,44,45]. Therefore, when studying the stress-strain state of the tank wall’s dent zone, it was decided that we would conduct experimental studies of the tank wall using fragment models with imperfections, on the basis of conducting theoretical studies and developing an engineering calculation method and constructive measures to reduce the stress concentration in the dent zone. 

#### 3.1.1. The Nature of the Stress-Strain State of Various Shapes’ Dent Zones

At the first stage of the experimental study, the stress-strain state of the dent zone in the model wall was assessed in order to study the stress distribution in the specified zone and establish the stress concentration coefficient’s dependence on the dent’s geometric dimensions and shape. 

**Figure 5:**
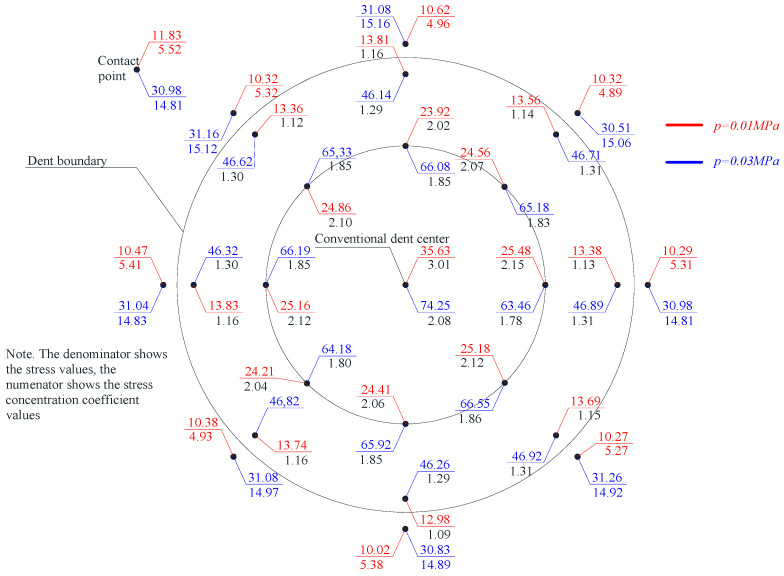
Distribution of hoop stresses at characteristic dent zone points in the wall of the model M1-A.

**Figure 6:**
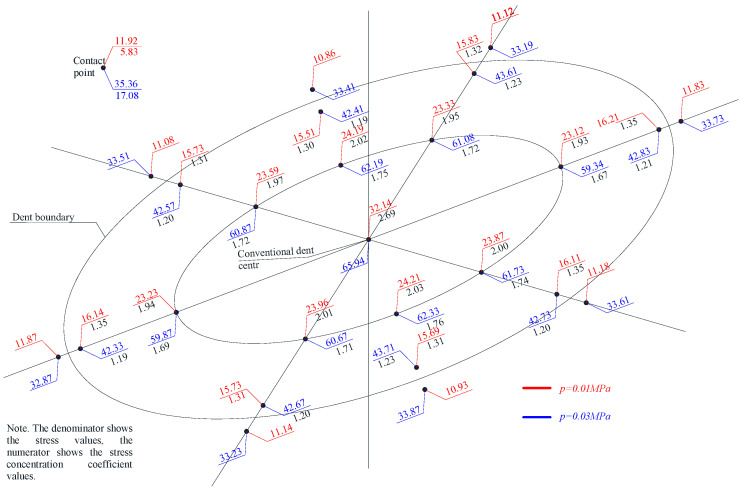
Distribution of hoop stresses at characteristic dent zone points in the wall of the model M1-B.

**Figure 7:**
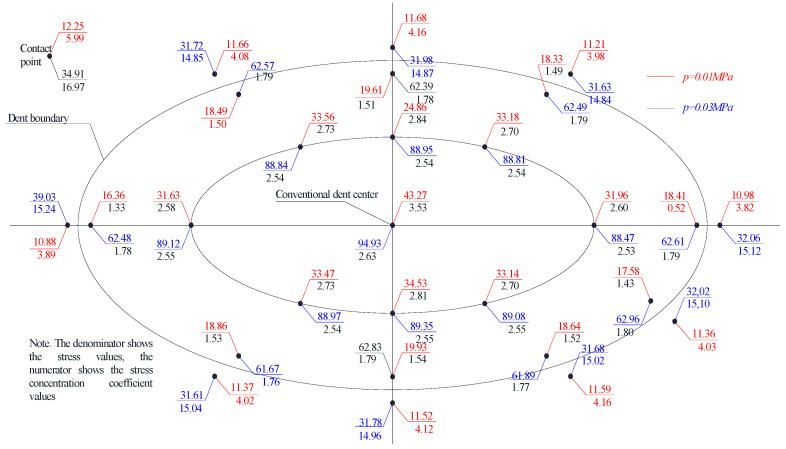
Distribution of hoop stresses at characteristic dent zone points in the wall of the model M2-A.

**Figure 8:**
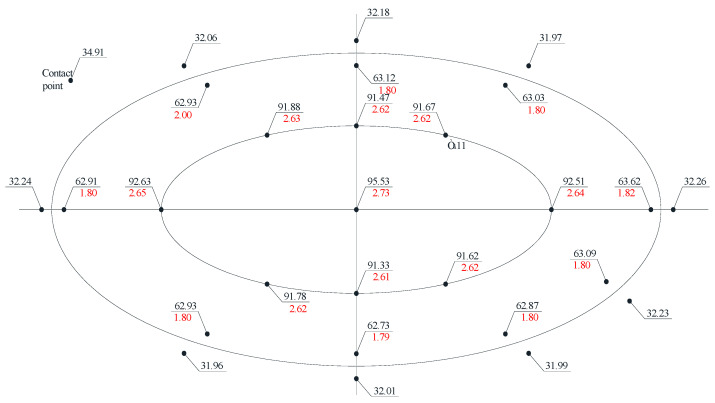
Distribution of hoop stresses at the characteristic dent zone points at the moment of the wall snapping out in the model M2-A.

Considering that hoop stresses exceed longitudinal stresses in cylindrical tanks, special attention was paid to the distribution of hoop stresses in the dent zone. The hoop stress experimental values’ distribution fields in the dent zones of models M1-A, M1-B and M2-A are presented in Figure 5, Figure 6, Figure 7 and Figure 8. 

The experimental stress values were determined by measuring the relative strains, ε_x_ and ε_y,_ at characteristic points on the wall surface and calculated using Equations (1) and (2). 

The nominal hoop and longitudinal stresses at the characteristic control points of the model wall’s defect-free zone were: in the model M1-A, at a pressure of 10 kPa–11.8 MPa and 5.52 MPa, respectively, at a pressure of 30 kPa–35.61 MPa and 17.21 MPa; in the model M1-B, at a pressure of 10 kPa–11.92 MPa and 5.83 Mpa, at a pressure of 30 kPa–35.36 Mpa and 17.08 Mpa; and in the model M2-A, at a pressure of 10 kPa–12.25 MPa and 5.99 MPa, respectively, at a pressure of 30 kPa–34.91 MPa and 16.97 MPa, respectively. 

The obtained nominal stress values are in full agreement with the calculated membrane stresses determined by the formulae for hoop stresses—*pD/2t*; for longitudinal stresses—*pD/4t*; where *p*—the pressure in the cylindrical shell and *D* and *t*—the shell diameter and thickness. 

The results of an assessment and analysis of the model wall’s stress-strain state in the dent zone showed that a local defect in the form of a dent on the model wall that is loaded with internal pressure is a local stress concentrator, and this fully confirms the assumption of local stress disturbances in the defect zone. 

The stress distribution nature, in Figure 5, Figure 6, Figure 7 and Figure 8, shows that there is an unloading zone along the defect boundaries on the dent located outside, where the local stresses are lower than the nominal ones. So, the decrease in these stresses in the unloading zones compared to the nominal values were up to 1.2 times lower in the model M1-A, in the model M1-B—up to 1.06 times lower, and in the model M2-A—up to 1.15 times lower, which was also observed in other studies [46,47,48,49]. 

It was found that the greater the dent depth and the smaller it was in size, then its unloading would be greater. The decrease in the stresses on the outer zones of the dent boundary is explained by the bursting effect on the part of the dent that is bent inside of the shell. At a distance from the dent zone boundaries, the hoop and longitudinal stresses in the model wall approach the nominal stresses, which indirectly confirms the correctness of the assumptions underlying the study. 

In the tested models, the largest local stresses were observed in the center zone of the dent, where the greatest depths were observed, and the hoop stresses exceeded the longitudinal ones. With an increase in the internal pressure in the model, the hoop stresses’ values in the dent’s center zone decreased, and their concentration was redistributed to the lateral boundary zones of the dent. This is explained by the bending of the dent and a decrease in the dent depth, with an increase in the load and redistribution of the stresses in the defect zone. 

In the dent zones in the walls of the models, M1-B and M2-B, where the dent contour smoothly passes into the shell’s surface, the maximum stress zone at the initial deformation stage shifts to the middle of the dent. The effect of the wall snapping out in the dent zone was not observed when the maximum pressure reached 30 kPa. With an increase in the load at the dent’s boundaries, the stresses were approximately equal, which is explained by the fact that the dent contours in these models are not sharply expressed and have a smooth character. 

When testing the model M2-A, when an internal pressure of 26 kPa was reached, the effect of the wall snapping out in the dent zone was observed, which was accompanied by a popping and a sharp outward bending of the central dent zone. 

The moment during which the wall snapped out in the dent zone, seen in Figure 8, was accompanied by the transition of the zones of maximum stresses and deformations to the side contour points, with a sharp increase in the stresses at the dent’s boundary zone points. In the middle of the defect, the dent zone popping was accompanied by instantaneous unloading, which is explained by a decrease in the dent depth as a result of the dent’s outward bending. 

It is easy to see that the determining influence on the stress concentration in the dent zone is exerted by the dent depth and the nature of the dent boundary transition line to the shell wall. 

**Figure 9:**
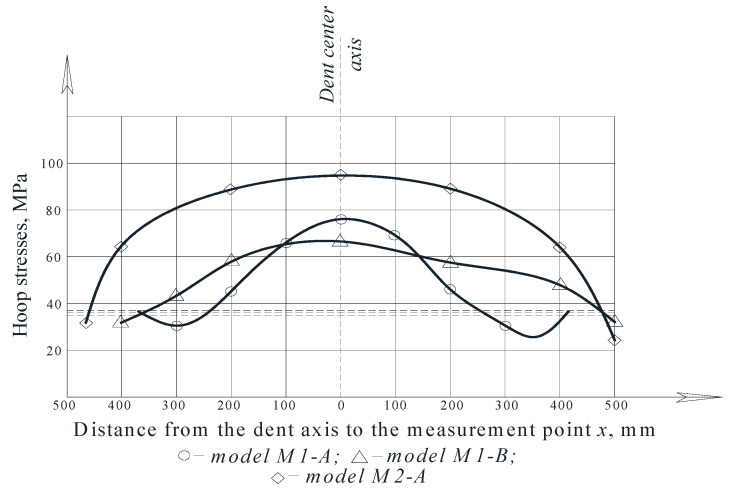
Diagrams of the hoop stresses in the dent zone [33].

The curves of the hoop stress diagram in Figure 9 indicate the characteristic stress concentration that was observed in the central dent zone. 

Note that the stress values in the dent zone are largely determined by its depth and geometric dimensions. The maximum stresses were achieved in the central dent zone in the model M2-A, which amounted to 91.93 MPa. 

#### 3.1.2. The Nature of the Movements of the Dent Zone in the Model When Loaded with Internal Pressure

In accordance with the tasks assigned to the experiments, measurements of the movement of the wall at the characteristic points of the dent zone were made. 

**Figure 10:**
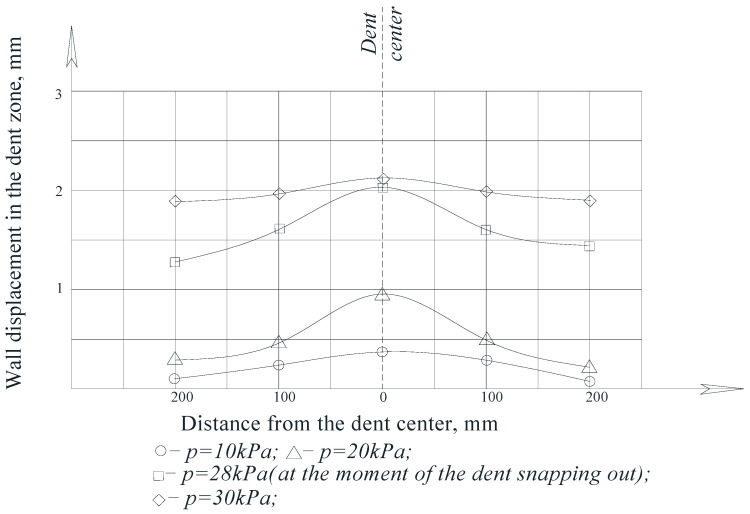
The wall displacements of the model, M1-A, at the characteristic spherical dent zone points [33,34].

**Figure 11:**
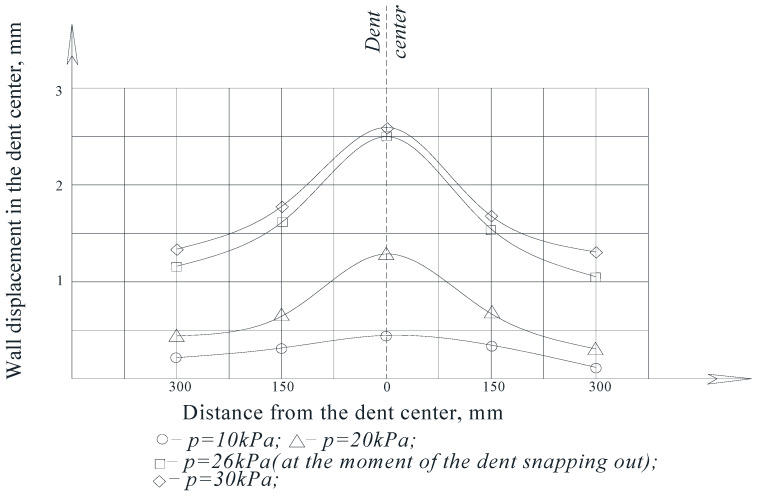
The wall displacements of the model, M2-A, at the characteristic elliptical dent zone points in the direction of the large ellipse radius.

The results of measuring the wall movements at the characteristic points of the dent zones at different values of internal pressure are shown in the form of diagrams in Figure 10 and Figure 11. 

As can be seen from the figures, when the models were loaded with internal pressure, the depth of the dent decreased as the pressure in the model increased.

The greatest wall movements were observed in the area of the center of the dent, and the smallest movements were observed in the areas closer to their borders.

**Figure 12:**
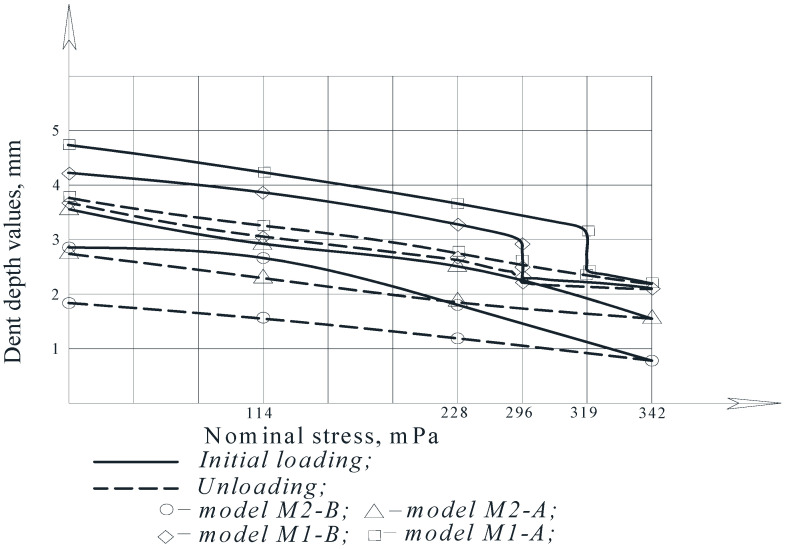
Changes in the value of the dent center depth value, *f*, depending on the nominal stresses σ_*n*_ [34].

Graphs of the changes in the depth of the dents that were under the nominal stresses in the model wall during initial loading and unloading are presented in Figure 12.

With an increase in pressure in the model, an irreversible decrease in the dent depth occurs, the degree of reduction of which is directly proportional to the applied load.

For comparison, Figure 10 shows the dependence of the residual dent depth, *f*_0_, on the nominal stresses, *σ_n_*—calculated by the formula [50,51]:

(4) $$\frac{f}{f_{0}}=1-0.6\frac{\sigma_{n}}{\sigma_{B}}$$

where *σ_n_ = pR/t*—the nominal stress in the model wall; *p*—an internal pressure; *t*—the wall thickness; and *σ_B_*—the limit of temporary resistance of the model wall material.

It was established that the dependence of the residual depth in the dent center, *f*_0_, on the nominal stresses, *σ_n_*, described by relation (4) does not agree well with the experimental data. It is noted that this is due to the significant deformability of the tank wall associated with its thinness. 

An analysis of the graphs of the model wall displacements in the dent zone shows that for tanks with significant wall deformability, a transformed formula should be used in the form:

(5) $$\frac{f}{f_{0}}=1-0.4\frac{\sigma_{n}}{\sigma_{B}}$$

In the process of testing on models M1-A and M2-A, the phenomenon of the snapping out (popping) of the wall in the dent zone was recorded, which corresponds to the results of [48]. According to this work, the phenomenon of a wall snapping out can be observed when the ratio of the dent depth *f*, to the shell wall thickness, *t*, is equal to *f/t* > 3.5.

If the above model facilitates some understanding about the phenomenon of popping, then it seems impossible to study the stress-strain state in the dent zone in the tank wall using this kind of analogy. 

In the model M1-A with a spherical dent of *f* = 4.32 mm deep (Figure 12), the phenomenon of the wall snapping out in the dent zone was observed at an internal pressure of 28 kPa. At the same time, the dent depth decreased to 2.06 mm, which is more than half of the initial depth and 55% of the established dent depth. 

In the model M2-A with an elliptical dent that was 4.86 mm deep, this phenomenon was observed at a pressure of 26 kPa. The initial dent depth was decreased to 2.34 mm.

In the case of a spherical dent (the model M1-A), upon unloading, the wall returned to its original position after snapping out; and in the case of an elliptical dent (the model M2-A), after snapping out during unloading, the dent did not return to its original position. 

#### 3.1.3. Assessment of the Stress Concentration in the Dent Zone of the Model

An experimental study of the stress state of the dent zone in the model tank wall substantiated the assumption of significant stress concentrations in the dent zone and pointed to the decisive influence of its geometric dimensions and, especially, its depth on the stress concentration in the dent zone. 

**Figure 13:**
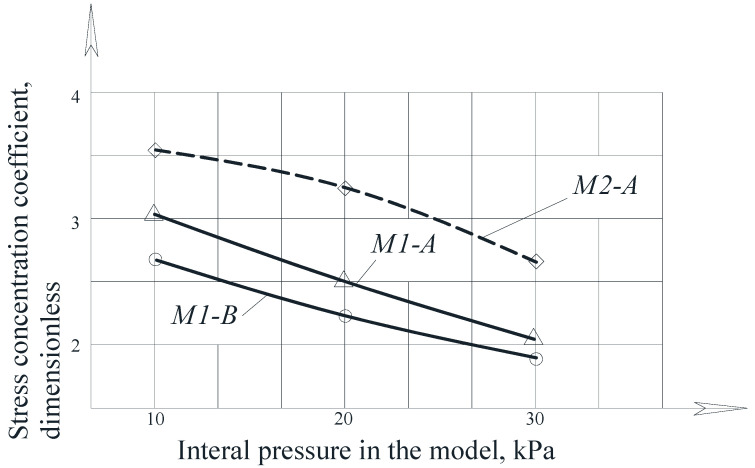
Dependence of the stress concentration coefficient in the dent zone on the internal pressure in the model [33,34].

The experimental dependences of the stress concentration coefficient in the dent zone on the internal pressure are presented in Figure 13. 

**Figure 14:**
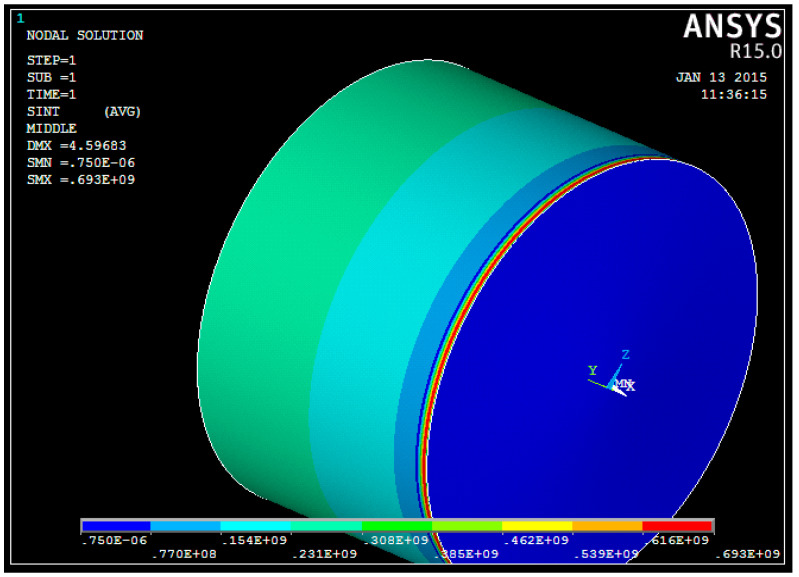
The results of calculating the equivalent stresses of the middle tank surface with a constant wall thickness [42].

An analysis of the graphs in Figure 14 shows that with increasing pressure, the stress concentration coefficient in the dent center decreases. Thus, in the models M1-A and M1-B, this decrease was 30% and 31%, respectively, and in the model M2-A it was 25%. This decrease is explained by the straightening of the dents and a decrease in their depth with an increase in the internal pressure in the model. 

However, it was noted that with increasing pressure, the intensity of the central dent zone decreased, and the region of the highest stress shifted to the dent boundaries. This fact also affected the stress concentration coefficient values. 

When analyzing the distribution of the hoop stresses at the characteristic dent zone points seen in Figure 3, Figure 4 and Figure 5, it is easy to notice a decrease in the stress concentration in the central dent zone and some increase in its border zones. 

The distribution of hoop stresses at the characteristic dent zone points in Figure 6 shows that at the moment the model wall snaps out in the dent zone, a sharp decrease in the stress concentration coefficient is observed with an increase in the stress concentration in zones that are close to the dent boundary and this stress transitions to the main shell wall. 

It should also be noted that in models with dents with characteristic bends along their boundaries, the stress concentration coefficients in the dent edge zone are much higher than in models where the dent boundaries smoothly pass into the main shell wall. This is probably because the bending ribs formed along the dent boundaries impart a certain rigidity to this zone, limit the wall deformation in the defect zone, and lead to an increase in the stress in this zone. During loading by internal pressure, smooth bends along the dent boundaries are deformed together with the wall zone along the outer dent boundaries, which leads to a smoother stress diagram in this zone. 

However, attention should be paid to significant stress concentrations in the dent zone, and the complex zone loading nature and its dependence on a number of factors: structure, operation, the dent shape and geometry, the dent formation placement, the presence or absence of the effect of the wall snapping out, wall thickness and shell radius. 

The conducted studies made it possible to qualitatively and quantitatively reveal the nature of stress distribution in the cylindrical shell wall’s dent zone and its dependence on the internal pressures at play, the dent shape and size, the wall thickness and the shell radius. 

### 3.2. Theoretical Study of Stress Concentration in the Dent Zones in the Tank Wall

#### 3.2.1. The Order and Verification of the Numerical Simulation Results of the Stress-Strain State of Tanks with Dents in the Wall

The study of the defect zone stress-strain state in dents in the cylindrical shell wall was a difficult task. In this regard, multilevel mathematical models of the structure and different wall thicknesses were used in the modeling technique [42]. 

An analysis [42] of the results of calculating the equivalent stresses presented in Figure 14 shows that the tank bottom is not loaded. The most loaded structure is the tank body. There is a significant contribution of hoop stresses, σ_θ_, to the overall stress-strain state of the tank body. The values of radial and longitudinal stresses are extremely small. The hoop stresses, σ_θ_, in the circumferential and longitudinal directions are constant. 

Table 2 presents the results of comparing the equivalent, σ*_i_*, and hoop, σ_θ_, stresses of the middle shell surface depending on the longitudinal coordinate *x*.

**Figure 15:**
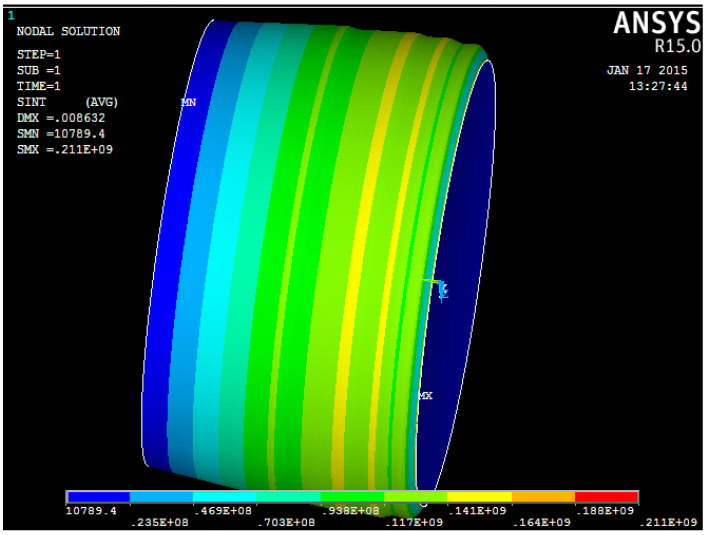
The results of calculating the equivalent stresses of the middle tank surface with a variable wall thickness [42].

Additionally, at this stage of numerical research, the stress-strain state of a variable thickness tank wall was considered in accordance with Figure 15 [42]. 

As can be seen from Table 2 and Table 3, the results of the hoop stress calculations obtained using the ANSYS software package and the analytical solution are similar. This indicates that to assess the stress-strain state of the cylindrical tank wall with a variable thickness, it is possible to use the relations for a cylindrical shell with a constant wall thickness [42]. 

#### 3.2.2. Stress-Strain State of the Cylindrical Tank Wall with a Dent

The geometric shapes of the dents in the tank wall are varied (spherical, diamond-shaped, elliptical, etc.). Considering the fact that dents of various geometric shape can be idealized into a spherical shape, it is helpful to consider spherical dents in terms of radius, *r*_B_, and depth, *f*.

Numerical studies were carried out using the ANSYS software package. A typical vertical cylindrical tank with a volume of 3000 m^3^ was considered. 

It is shown that, far from the stress concentrator (dent), the analytical formula can be used to calculate the stress-strain state. For this, we used the results of a calculation of the hoop stresses, σ_θ_, far from the dent, presented in Table 4. 

The first column of Table 4 shows the longitudinal coordinate values of the design points of the tank. The second column shows the values of the hoop stresses, σ_θ_, calculated using ANSYS, and the third column shows the results of a calculation of the hoop stresses using the expression obtained in [42]. 

The results of a calculation of the hoop stresses, σ_θ_, according to the expression, and using the ANSYS software package, are similar, which proves the possibility of using the analytical formula to calculate the equivalent stresses far from the dent. 

#### 3.2.3. Numerical Studies of Stress Concentration in the Dent Zone in the Tank Wall

Experimental and numerical assessments of the dent zone stress-strain state in the tank wall have proved the assumption of significant stress concentrations in the dent zone. There is a determining influence on the stress concentration in the dent zone by its geometric dimensions and especially, its depth [42].

The results of the calculation of the stress concentration coefficients using the ANSYS software package for various values of dimensionless dent parameters: dimensionless radius—ξ; and dimensionless depth of the dent—ς, are presented in Table 5. 

Based on the results of a numerical study, graphical dependences of the stress concentration coefficient, *K*_σ_, on the dimensionless dent depth, ς, were constructed for various values of the dimensionless dent radius, ξ [42]. 

The obtained dependences of the stress concentration coefficient, *K*_σ_, on the dimensionless dent depth, ς, and the dimensionless dent radius, ξ, confirmed the decisive influence on the stress concentration in the dent zone of its depth, *f*. At the same time, the obtained dependences of the stress concentration coefficient on the dent parameters are important from the point of view of obtaining an engineering-based, empirical calculation formula. 

#### 3.2.4. Determination of Stress Concentration Coefficients in the Dent Zone

The results of calculating the stress concentration coefficients for various sizes of spherical dents using the obtained dependence are presented in Table 6 [42]. 

The data in Table 6 show that the calculated values of the stress concentration coefficient determined by a numerical calculation using ANSYS and by the analytical expression [40] are quite similar and do not exceed 2% of the indicator, which indicates the correctness of the obtained analytical expression. 

The resulting empirical formula can be used for engineering calculations of stress concentration coefficients for other tanks with other dent sizes. Based on nomograms were constructed to determine the stress concentrations on the geometric dent dimensions [42]. 

The resulting nomogram makes it possible, given the known geometric dent dimensions for a particular tank, to determine the stress concentration coefficients in the defect zone. 

## 4. Results and Discussion

Experimental tests of models of a body fragment with dents of various shapes and geometric sizes has established that during loading and unloading, an irreversible decrease in the dent depth occurs, which is directly proportional to the applied load. 

Thus, when studying models for the stress–strain state of the dent zone in the tank wall to understand the nature of the stress-strain state of the dent zone of various shapes (Table 1), it was found that the nature of stress distributions (Figure 5, Figure 6, Figure 7 and Figure 8) showed that there is an unloading zone along the boundaries of the defect on the outside of the dent, where local stresses are lower than the nominal ones. In the M1-A model, the decrease in these stresses in the unloading zone was up to 1.2 times lower; in the M1-B model—up to 1.06 times lower; and in the M2-A model—up to 1.15 times lower, which was also observed in other studies [52,53,54,55,56,57]. It should be noted that the determining influence on the stress concentration in the dent zone is exerted by the depth of the dent and the nature of the transition lines of the dent boundary along the shell wall. The maximum values of the dent stresses were located in the central part, that is, where the depth of the dent corresponds to the maximum, as seen in Figure 9.

When studying the nature of the movements of the dent zone experimentally, it was found that the earlier results presented in expression (4) in the works [58,59,60,61,62,63] are insufficient, since they do not fully agree with the results of the experiments. 

Due to the fact that during the experiments it was established that the wall was leached (popped) under certain loads, it is recommended to use the expression (5), which corresponds to the results of the work [47,48]. Thus, the most appropriate expression was experimentally determined.

Later, when assessing the stress concentration in the dent area, it was found that with increasing pressure, the stress concentration coefficient in the center of the dent decreased. So, in the M1-A and M1-B models, this decrease was 30% and 31%, respectively, and in the M2-A model it was 25%. This decrease is explained by the correction of dents and a decrease in their depth with an increase in internal pressure in the model, as shown in Figure 13.

In the study, additional theoretical studies were carried out in the form of a numerical modeling of a 3000 m^3^ tank. The main results of the numerical modeling are presented in the work [42], which was carried out earlier than this one. The study also presents analytical solutions that were compared with calculations carried out using the ANSYS software package, where the spread of values is from 2% to 10%. It is noted that depending on the location of the dent, its size and operating conditions, the stress concentration can reach up to 10–11 times that of the nominal values.

In general, it can be noted that the numerical evaluation of the stress-strain state of the dent zone in the tank wall has proved the assumption of significant stress concentrations in the dent zone and indicated a determining influence on the stress concentration in the dent zone of its geometric dimensions and especially its depth, which was also established experimentally. 

In the course of the study, the empirical formula obtained, [42], can be used for engineering calculations of stress concentration coefficients of other tanks with other dent sizes. 

Nomograms were constructed to determine the stress concentration from the geometric dimensions of the dent, and the radius and thickness of the tank wall in accordance with [42].

However, it should be noted that the study was conducted for an idealized, spherical dent in the tank wall. The authors, realizing the limitations of the application of a method to determine the dependence of the stress concentration on the size of the dent within the study of engineering, will continue researching in the direction of a more advanced mathematical model for determining the stress concentration in the defect zone. 

This study is a continuation of the studies [10,11,23,40,63,64,65,66,67,68,69,70,71,72,73,74,75] conducted as part of the study of the actual operation of vertical cylindrical tanks for oil and petroleum products. In the future, there is a need for field studies of the stress-strain state of tank structures with various geometric shape imperfections. At the same time, the issues of the normalization of the dimensions of geometric imperfections in the shape of the tank wall and the development of methods to eliminate or strengthen them remain important.

## 5. Conclusions

Theoretical experimental studies were carried out in this work, in which identical results were obtained with a spread of difference from 2% to 10%, which is a satisfactory indicator of the correctness of the hypotheses. By testing the fragments of a cylindrical tank, the most suitable expression (5) was found, which could take into account the leaching of the tank wall, resulting in a decrease in the stress concentration index, which was not previously taken into account. It was also experimentally established that the geometric dimensions of the dent, especially its depth, have a great influence on the stress–strain state of the tank.

At the same time, the results of these theoretical studies fully confirmed the results obtained experimentally, where the main conclusion was the statement about the influence of the geometric dimensions of the dents on the stress concentration index. 

In the conducted study, the results were obtained as follows: a suitable expression was experimentally established, taking into account the leaching of the reservoir under internal loading; polynomials were also previously obtained for determining stress concentration coefficients [42]; and the main reason for the increase in stress concentrations in the defect in the form of a dent was revealed.

## Figures and Tables

**Table 1:** The shape and geometric dimensions of dents in the model body [33,34].

Model	Model Surface	Dent Shape	Dent Radius, mm		Dent Depth, mm	
			Small	Large	Initial	Steady-State
M 1	A	spherical	252	-	4.32	3.68
	B	elliptical	185	820	3.53	2.71
M 2	A	elliptical	175	830	4.86	3.73
	B	elliptical	190	640	2.84	1.84

**Table 2:** The results of comparing the equivalent σ*_i_* and hoop σ_θ_ stresses of the middle shell surface.

Design Point Coordinate *x*, m	Equivalent Stresses, σ*_i_*, Pa	Hoop Stresses, σ_θ_, Pa
1.0728	0.14251 × 10^9^	0.118 × 10^8^
1.2516	0.13859 × 10^9^	0.118 × 10^8^
2.2052	0.15865 × 10^9^	0.158 × 10^8^
2.5032	0.15800 × 10^9^	0.158 × 10^8^
3.2184	0.19150 × 10^9^	0.19 × 10^8^
3.4568	0.19107 × 10^9^	0.19 × 10^8^
3.8144	0.19007 × 10^9^	0.19 × 10^8^
4.4104	0.18994 × 10^9^	0.19 × 10^8^
4.7680	0.18997 × 10^9^	0.19 × 10^8^
5.1256	0.19005 × 10^9^	0.19 × 10^8^
5.3640	0.18952 × 10^9^	0.19 × 10^8^
6.1984	0.24439 × 10^9^	0.238 × 10^8^
6.4368	0.23993 × 10^9^	0.238 × 10^8^
6.6752	0.23778 × 10^9^	0.238 × 10^8^
8.8208	0.23745 × 10^9^	0.238 × 10^8^
10.728	0.23745 × 10^9^	0.238 × 10^8^

**Table 3:** The results of comparing the calculated values of the equivalent, σ*_i_*, and hoop, σ_θ_, stresses in the tank wall.

Design Point Coordinate *x*, m	Equivalent Stresses, σ*_i_*, Pa	Hoop Stresses, σ_θ_, Pa
1.3112	0.12294 × 10^9^	1.11113 × 10^8^
1.7880	0.14321 × 10^9^	1.41493 × 10^8^
2.5032	0.13125 × 10^9^	1.31505 × 10^8^
3.2184	0.14962 × 10^9^	1.45821 × 10^8^
4.0528	0.13178 × 10^9^	1.31838 × 10^8^
5.1256	0.11389 × 10^9^	1.13860 × 10^8^
6.1984	0.12359 × 10^9^	1.19853 × 10^8^
7.1520	0.99855 × 10^8^	9.98776 × 10^7^
8.3440	0.74893 × 10^8^	7.49082 × 10^7^
8.8208	0.64907 × 10^8^	6.49204 × 10^7^
9.5360	0.49928 × 10^8^	4.99388 × 10^7^

**Table 4:** The results of calculating the hoop stresses, σ_θ_, in the tank wall using the ANSYS software package and according to [42].

Design Point Coordinate *x*, m	Hoop Stresses, σ_θ_, Pa, According to ANSYS	Hoop Stresses, σ_θ_, Pa, According to [42]
4.6098	0.116 × 10^9^	0.122 × 10^9^
5.2721	0.166 × 10^9^	0.114 × 10^9^
5.1933	0.138 × 10^9^	0.112 × 10^9^
5.6411	0.121 × 10^9^	0.105 × 10^9^
7.1527	0.9825 × 10^8^	0.9986 × 10^8^
8.5363	0.697 × 10^8^	0.709 × 10^8^

**Table 5:** The results of calculating the stress concentration factor, *K*_σ_, in the dent zone in the cylindrical tank wall.

Dimensionless Radius of the Dent ξ	Dimensionless Depth of the Dent ς	The Dent Radius *r_b_*, m	The Dent Depth *f*, m	The Stress Concentration Coefficient, *K*_σ_
1	2	3	4	5
2	4	0.3899	0.016	4.937
2	7	0.3899	0.028	5.3121
2	10	0.3899	0.04	5.406
2	13	0.3899	0.052	5.371
2	15	0.3899	0.06	5.4891
2	18	0.3899	0.072	5.3855
3	4	0.585	0.016	5.672
3	7	0.585	0.028	6.383
3	10	0.585	0.04	6.7322
3	13	0.585	0.052	6.852
3	15	0.585	0.06	6.978
3	18	0.585	0.072	6.906
4	4	0.7797	0.016	6.135
4	7	0.7797	0.028	7.391
4	10	0.7797	0.04	7.405
4	13	0.7797	0.052	7.944
4	15	0.7797	0.06	7.561
4	18	0.7797	0.072	7.714
5	4	0.97	0.016	6.29
5	7	0.97	0.028	8.136
5	10	0.97	0.04	10.02
5	13	0.97	0.052	8.34
5	15	0.97	0.06	8.757
5	18	0.97	0.072	9.0
6	4	1.17	0.016	6.7493
6	7	1.17	0.028	8.4853
6	10	1.17	0.04	10.54
6	13	1.17	0.052	9.204
6	15	1.17	0.06	9.4476
6	18	1.17	0.072	9.598
7	4	1.365	0.016	8.288
7	7	1.365	0.028	8.4469
7	10	1.365	0.04	11.12
7	13	1.365	0.052	10.02
7	15	1.365	0.06	10.09
7	18	1.365	0.072	10.622
8	4	1.5595	0.016	9.6269
8	7	1.5595	0.028	8.4231
8	10	1.5595	0.04	9.849
8	13	1.5595	0.052	10.451
8	15	1.5595	0.06	10.74
8	18	1.5595	0.072	11.013
9	4	1.7544	0.016	10.75
9	7	1.7544	0.028	8.567
9	10	1.7544	0.04	10.30
9	13	1.7544	0.052	11.52
9	15	1.7544	0.06	11.34
9	18	1.7544	0.072	11.799

**Table 6:** The results of comparing the values of stress concentration coefficients calculated by the numerical method and using the formula.

Dimensionless Radius of the Dent, ξ	Dimensionless Depth of the Dent, ς	The Dent Radius, *r_b_*, m	The Dent Depth, *f*, m	Stress Concentration Coefficient by Numerical Calculation, *K*_σ_	Stress Concentration Coefficient Calculated by the Formula [42], *K*_σ_
2	4	0.3899	0.016	4.937	4.9341
2	7	0.3899	0.028	5.3121	5.3268
2	10	0.3899	0.04	5.406	5.3736
2	13	0.3899	0.052	5.371	5.4142
2	15	0.3899	0.06	5.4891	5.4625
2	18	0.3899	0.072	5.3855	5.3892
3	4	0.585	0.016	5.672	5.6699
3	7	0.585	0.028	6.383	6.3939
3	10	0.585	0.04	6.7322	6.7083
3	13	0.585	0.052	6.852	6.8840
3	15	0.585	0.06	6.978	6.9584
3	18	0.585	0.072	6.906	6.9088
4	4	0.7797	0.016	6.135	6.1521
4	7	0.7797	0.028	7.391	7.3004
4	10	0.7797	0.04	7.405	7.6036
4	13	0.7797	0.052	7.944	7.6784
4	15	0.7797	0.06	7.561	7.7231
4	18	0.7797	0.072	7.714	7.6898
5	4	0.97	0.016	6.29	6.2459
5	7	0.97	0.028	8.136	8.3655
5	10	0.97	0.04	10.02	9.5133
5	13	0.97	0.052	8.34	9.0134
5	15	0.97	0.06	8.757	8.3414
5	18	0.97	0.072	9.0	9.0566
6	4	1.17	0.016	6.7493	6.7114
6	7	1.17	0.028	8.4853	8.6836
6	10	1.17	0.04	10.54	10.104
6	13	1.17	0.052	9.204	9.7865
6	15	1.17	0.06	9.4476	9.0918
6	18	1.17	0.072	9.598	9.6508
7	4	1.365	0.016	8.288	8.2487
7	7	1.365	0.028	8.4469	8.6508
7	10	1.365	0.04	11.12	10.669
7	13	1.365	0.052	10.02	10.618
7	15	1.365	0.06	10.09	9.7212
7	18	1.365	0.072	10.622	10.672
8	4	1.5595	0.016	9.6269	9.6158
8	7	1.5595	0.028	8.4231	8.4732
8	10	1.5595	0.04	9.849	9.7329
8	13	1.5595	0.052	10.451	10.600
8	15	1.5595	0.06	10.74	10.643
8	18	1.5595	0.072	11.013	11.022
9	4	1.7544	0.016	10.75	10.750
9	7	1.7544	0.028	8.567	8.5492
9	10	1.7544	0.04	10.30	10.332
9	13	1.7544	0.052	11.52	11.475
9	15	1.7544	0.06	11.34	11.369
9	18	1.7544	0.072	11.799	11.799

## Data Availability

Data sharing is not applicable to this article.
